# Porcine Stomach Smooth Muscle Force Depends on History-Effects

**DOI:** 10.3389/fphys.2017.00802

**Published:** 2017-10-18

**Authors:** André Tomalka, Mischa Borsdorf, Markus Böl, Tobias Siebert

**Affiliations:** ^1^Department of Sport and Motion Science, University of Stuttgart, Stuttgart, Germany; ^2^Department of Mechanical Engineering, Institute of Solid Mechanics, Braunschweig University of Technology, Braunschweig, Germany

**Keywords:** force depression, force enhancement, force-length relation, force-velocity relation, gastric contraction-behavior, muscle properties, smooth muscle tissue, gastric motility

## Abstract

The stomach serves as food reservoir, mixing organ and absorption area for certain substances, while continually varying its position and size. Large dimensional changes during ingestion and gastric emptying of the stomach are associated with large changes in smooth muscle length. These length changes might induce history-effects, namely force depression (FD) following active muscle shortening and force enhancement (FE) following active muscle stretch. Both effects have impact on the force generating capacity of the stomach, and thus functional relevance. However, less is known about history-effects and active smooth muscle properties of stomach smooth muscle. Thus, the aim of this study was to investigate biomechanical muscle properties as force-length and force-velocity relations (FVR) of porcine stomach smooth muscle strips, extended by the analysis of history-effects on smooth muscle force. Therefore, in total *n* = 54 tissue strips were dissected in longitudinal direction from the ventral fundus of porcine stomachs. Different isometric, isotonic, and isokinetic contraction protocols were performed during electrical muscle stimulation. Cross-sectional areas (CSA) of smooth muscles were determined from cryo-histological sections stained with Picrosirius Red. Results revealed that maximum smooth muscle tension was 10.4 ± 2.6 N/cm^2^. Maximum shortening velocity (*V*_*max*_) and curvature factor (*curv*) of the FVR were 0.04 ± 0.01 [optimum muscle length/s] and 0.36 ± 0.15, respectively. The findings of the present study demonstrated significant (*P* < 0.05) FD [up to 32% maximum muscle force (*F*_*im*_)] and FE (up to 16% *F*_*im*_) of gastric muscle tissue, respectively. The FE- and FD-values increased with increasing ramp amplitude. This outstanding muscle behavior is not accounted for in existing models so far and strongly supports the idea of a holistic reflection of distinct stomach structure and function. For the first time this study provides a comprehensive set of stomach smooth muscle parameters including classic biomechanical muscle properties and history-dependent effects, offering the possibility for the development and validation of computational stomach models. Furthermore, this data set facilitates novel insights in gastric motility and contraction behavior based on the re-evaluation of existing contractile mechanisms. That will likely help to understand physiological functions or dysfunctions in terms of gastric accommodation and emptying.

## Introduction

Smooth musculature is located in the walls of various hollow organs, like the urinary bladder, the intestine, and the stomach; transporting several substances (e.g., fluids, bolus, and nutrients) by muscle contraction. The stomach is part of the gastrointestinal tract, connecting the gullet (esophagus) to the duodenum, while mainly serving as a mixing area and holding reservoir. Furthermore, to accommodate a large amount of food, the position, and size of the stomach varies continually—yielding the digestive organ to the most distensible portion of the gastrointestinal tract (Tortora and Nielsen, 2013). Hence, it's biomechanical properties are of great functional importance (Zhao et al., 2005).

To enable the variability of functions of smooth gastric muscle, an elegant adjustment of varying contraction-types [e.g., concentric (active shortening), isometric (under constant length), and eccentric contractions (active lengthening)]—ensuring tonic and peristaltic contraction-behavior—is necessary (Schulze-Delrieu et al., 1998; Pal et al., 2004, 2007). To better understand stomach motility and function, knowledge about the influence of muscle length, velocity, activation level, and history-dependent effects (Hill, 1938; Abbott and Aubert, 1952; Huxley and Hanson, 1954; Gordon et al., 1966; Ebashi and Endo, 1968; Rode et al., 2016) on smooth muscle force is required. Due to the structural and mechanical similarity of the porcine to the human stomach (Zhao et al., 2008; Jia et al., 2015), the examination of stomachs from pigs is of particular importance. However, appropriate studies examining active and passive muscle properties are rare. While only a handful of studies have been observed force-velocity relations (FVR) on guinea pigs (Moriya and Miyazaki, 1985) and toads (Warshaw, 1987), intensive research has been done on the relationship between muscle length and force production in a variety of vertebrate and invertebrate smooth muscles (Gordon and Siegman, 1971; Herlihy and Murphy, 1973). Nevertheless, there is a limited knowledge about the shape and the microstructural understanding of the entire force-length relation (FLR) of stomach smooth muscles (Siegman et al., 2013). Consequently, almost nothing is known about the classic biomechanical properties as force-length and FVRs in the porcine stomach (Gunst, 1986; Minekus and van Mastrigt, 2001).

The influence of history-dependent effects on stomach smooth muscle force is of special interest for developing a functional understanding of the peristaltic mode of operation. Since more than six decades it has been known that skeletal muscle force depends on history-effects (Abbott and Aubert, 1952). For example, force is enhanced in the isometric phase following active stretching (force enhancement, FE) by up to 100% (Edman et al., 1982; Leonard and Herzog, 2010) and depressed following active shortening force depression (FD) by up to 20% (Herzog and Leonard, 1997; Siebert et al., 2015) compared with the corresponding isometric muscle force. Force generation during gastric distension as well as during and after gastric emptying is accompanied by large muscle length changes and, consequently, might be associated with FE and FD, respectively. Although there are a couple of studies reporting FD and FE for urinary bladder smooth muscle (Minekus and van Mastrigt, 2001; van Asselt et al., 2007; Menzel et al., 2017), to the best of our knowledge, there are no studies of history-effects in the porcine stomach.

Hence, the aims of this study were the determination and analysis of biomechanical muscle properties (force-length and FVRs) of intact, activated smooth muscle tissue from porcine stomach. Furthermore, this study represents the first *in-vitro* approach to examine the influence of history-dependent effects on gastric smooth muscle force and the functional interpretation thereof.

## Materials and methods

### Preparation and handling

Fresh porcine stomachs were chosen for this study due to their structural and mechanical similarity to the human stomach (Zhao et al., 2008; Jia et al., 2015). The stomachs were taken from 41 freshly killed female pigs (~6 months and 100 kg) of a local slaughterhouse. The experimental set-up, handling, and preparation of gastric smooth muscle tissue have been described earlier (Menzel et al., 2017).

**Figure 1:**
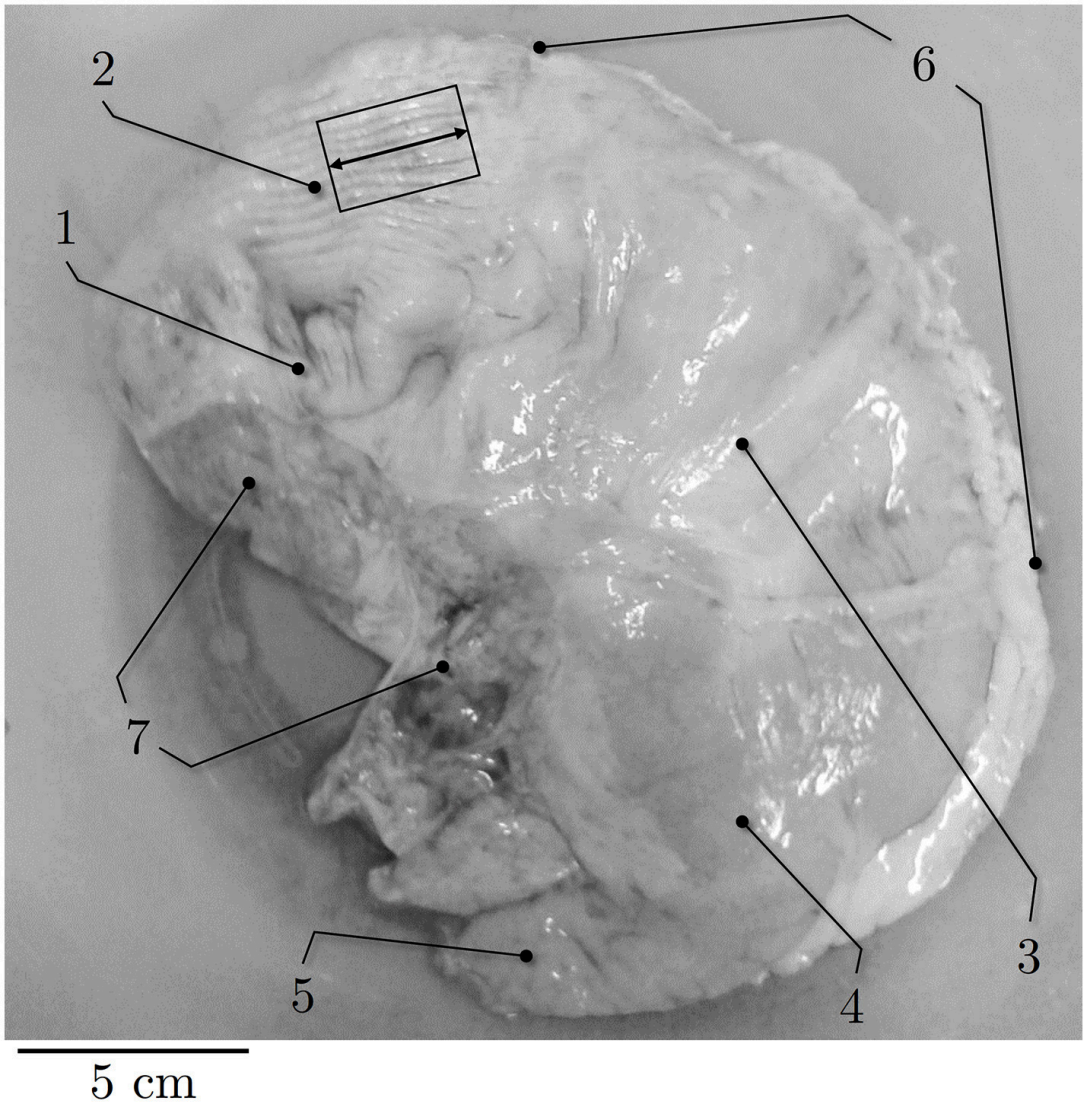
Representative picture of porcine stomach. Ventral view of external anatomy of a porcine stomach. (1) cardia, (2) fundus, (3) corpus, (4) antrum, (5) pylorus, (6) greater curvature, (7) smaller curvature. Black rectangle indicates longitudinal muscle strip dissected from the fundus. Longitudinal direction is marked by the black arrow.

Briefly, immediately after death a predefined piece of smooth gastric tissue (30 × 20 mm) was dissected from the ventral fundus of the proximal stomach (Figure 1) and separated from the tunica mucosa. Care was taken to prevent contact of the tissue strip with gastric acid to avoid smooth muscle degeneration. Afterwards, the preparation was preserved in an icebox in aerated (95% O_2_ and 5% CO_2_) Krebs solution (124 mM NaCl; 5 mM KCl; 2.5 mM CaCl_2_; 15 mM NaHCO_3_; 1.2 mM KH_2_PO_4_, 1.2 mM MgSO_4_, 10 mM C_6_H_12_O_6;_ pH 7.3 at 32°C) (Moriya and Miyazaki, 1982) at a constant temperature of 4°C for transportation. Subsequently, within 60–90 min postmortem, small tissue strips (0.66 ± 0.2 g) of 16 × 8 mm in longitudinal orientation (i.e., parallel to the direction of the gastric serosal fold, parallel with the greater curvature, Figure 1) were prepared in the laboratory from the predefined tissue sample of the fundus.

### Experimental set-up

The gastric strips were mounted vertically in an Aurora 805A *in-vitro* muscle apparatus between an alligator clip and two flexible cannula hooks on the bottom. The clamp was attached to a dual mode lever arm system (Aurora Scientific 305C-LR, force range: 10 N, force resolution: 1 mN). An initial passive baseline force of 5–10 mN was set by the test apparatus and the tissue strip length at this force, measured between clamp and hooks with a digital sliding caliper, was defined as the slack length (*L*_*s*_). The mean slack length was 13.42 ± 1.42 mm.

The tissue strips were electrically stimulated (Aurora Scientific 701C) with alternating pulses of 1 A, 100 Hz frequency, and 5 ms pulse width (van Mastrigt and Glerum, 1985; Menzel et al., 2017). All mechanical experiments were conducted at a constant temperature of 32 ± 0.1°C in aerated Krebs solution. At this temperature, the tissue samples proved very stable and able to withstand active protocols over an extended period of time (Essig et al., 1985; Tomalka et al., 2017). After an equilibration period of 30 min at *L*_*s*_, the strips were stimulated isometrically for about 15 s every 5 min until a steady state force [deviation <5% of maximum isometric force (*F*_*im*_)] was reached (Herrera et al., 2005).

### Determination of smooth muscle tissue properties

To determine the specific biomechanical muscle properties FLR, FVR, and history-dependent effects, isometric, isotonic, and isokinetic contractions have been performed in accordance with previous studies for smooth muscle tissue (Menzel et al., 2017; Seydewitz et al., 2017) and skeletal muscles (Böl et al., 2013; Siebert et al., 2015). Fifty-four (*n* = 54) tissue strips were used in total for the *in-vitro* dynamic parameter determination (Table 1). To investigate the active and passive FLR, a series of 18–19 isometric contractions (with length increments of 0.1 *L*_*s*_ in ascending order), starting from an initial sample length of 0.8 *L*_*s*_, have been conducted. Tissue strips were stretched up to passive forces of about 50% *F*_*im*_ to avoid muscle damage induced by excessive lengthening. At each length, the passive and maximum active muscle force were determined as the maximum force value and the passive force at the instant before muscle activation, respectively. Linear regression models following the equation*f*(*x*) = *mx* +*b*, with*x* = *L*/*L*_*S*_, were used to fit the ascending- and descending limb of the FLR. The muscle length at *F*_*im*_ was defined as the optimal muscle length (*L*_0_).

**Table 1:** Categorization of observed muscle properties from smooth porcine tissue strips. *n* is the number of samples.

**Strip number**	**S1–S14**	**S15–S37**	**S38–S42**	**S43**	**S44–S53**	**S54**	***n***
Force-length relation	x		x			x	20
Force-velocity relation		x		x		x	25
History-dependent effects			x	x	x	x	17

The FVR was identified by a series of about six isotonic contractions starting from *L*_0_ against forces in the range of 0.10–0.90 *F*_*im*_ (Till et al., 2008) in ascending order. The FVR followed the typical hyperbolic Hill equation (Hill, 1938) $f\left(v\right)=\left(v_{max}-v\right)/\left(v_{max}+\frac{v}{curv}\right)$, *v* < 0 for concentric contractions, with ν_*max*_ defined as maximum shortening velocity and $curv=\frac{a}{F_{im}}$ (where *a* is the asymptote of force; Hill, 1938). A recovery phase of 7 min between the experiments has been conducted over the entire experimental protocol for determination of force-length and FVRs.

**Figure 2:**
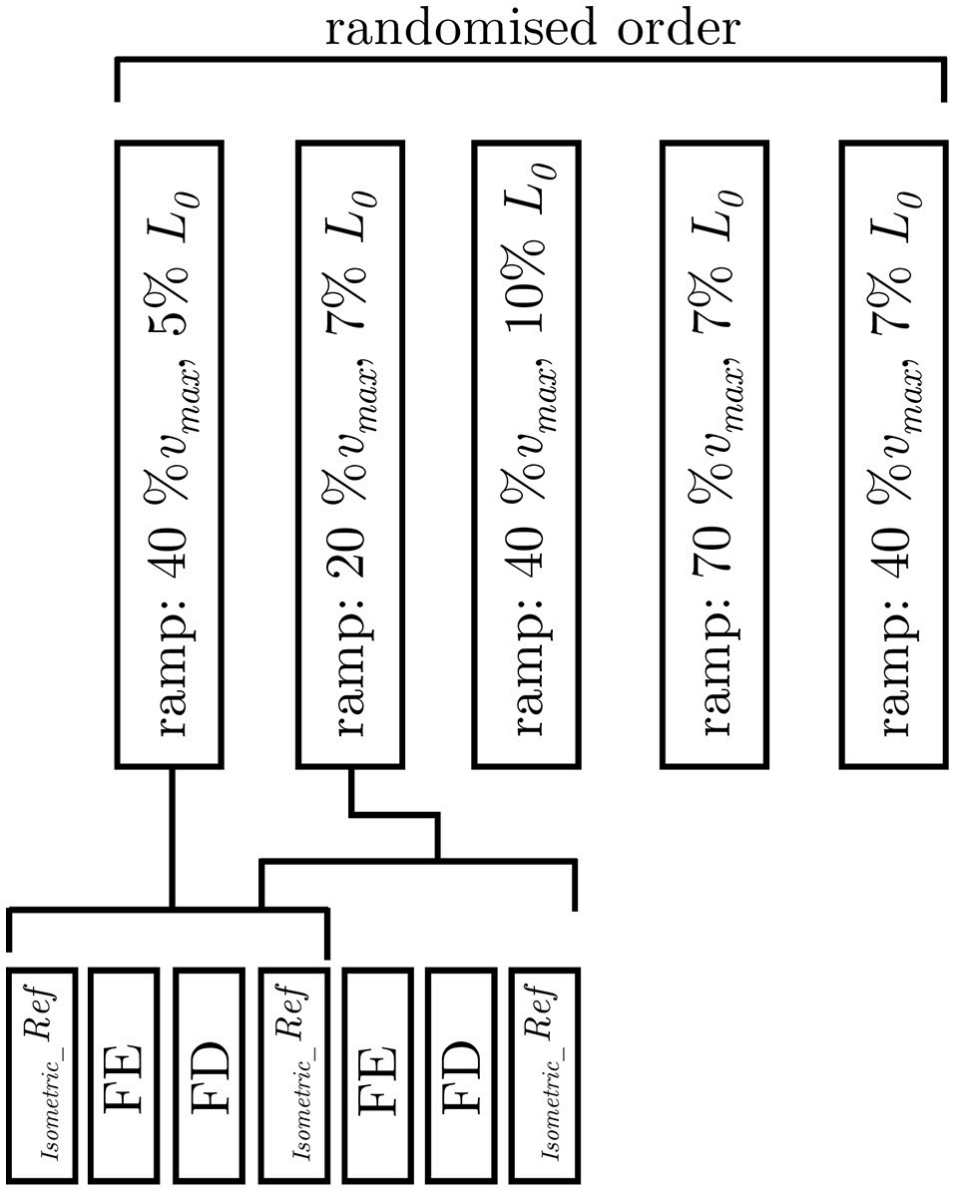
Experimental protocol. Examination of varying ramp parameters followed a pseudorandomised block design (upper trace). The applied ramp-velocity and ramp-length are normalized to the maximum shortening velocity (ν_*max*_) and optimum muscle length (*L*_0_), respectively. The lower trace illustrates the fixed protocol of muscle contractions consisting of isokinetic ramp experiments (FE: force enhancement, FD: force depression), and isometric reference contractions (_*Isometric*__*Ref*).

In order to investigate the impact of ramp length and velocity on history-dependent effects, isokinetic contractions (Herzog and Leonard, 1997; Menzel et al., 2017) have been applied to *n* = 17 tissue strips in dependence of varying ramp amplitudes at a given velocity (40% ν_*max*_) and in dependence of different velocities at a constant length amplitude (7% *L*_0_), respectively. The isokinetic ramps started after a period of pre-stimulation (around 14 s) under full activation until steady-state maximum isometric force, characterized by the development of a plateau, was reached. The stimulation continued for at least 14 s after the end of the ramp. Shortening and lengthening ramps for the determination of FD and FE in dependence of ramp length started at *L*_0_ ± 5, ± 7, and ± 10% *L*_0_, respectively, and were finished at *L*_0_. To examine the dependency of history-effects on speed, concentric and eccentric ramps from *L*_0_ ± 7% to *L*_0_ with different ramp velocities (20, 40, 70% ν_*max*_) were applied to the tissue strips. All ramp experiments have been carried out in randomized order (Figure 2). To calculate FE and FD, the difference between the redeveloped and the corresponding purely isometric force at the same length has been identified 10 s after the end of each ramp. The relaxation period during the entire history-block was 12 min to ensure an optimal recovery phase of force-generating processes (Gunst, 1986). The “cycling-protocol” by Brenner (1983) was utilized to conserve the structural and mechanical properties in maximally activated smooth gastric strips over an extended period of time as well as to reduce length-inhomogeneities of overlapping myofilaments.

To calculate force degradation, isometric reference contractions at *L*_0_ were performed before and after each of the ramp experiments. Plastic length adaptations of smooth muscle strips by dissolution and reformation of myosin filaments (Seow, 2005), occurring at periods of hours (Wang et al., 2001; Martinez-Lemus et al., 2004) or days (Arner et al., 1984; Zeidan et al., 2000; Naghshin et al., 2003) were not considered within this study, as the time period between the reference contractions was much shorter and muscle strips were held at a constant length (*L*_0_) during the experiments. During isokinetic ramp experiments, the isometric force in successive activations decreased at an average rate of 1.1% per activation. Data from preparations that produced an isometric force <6 N/cm^2^ have been rejected.

### Histological observations

**Figure 3:**
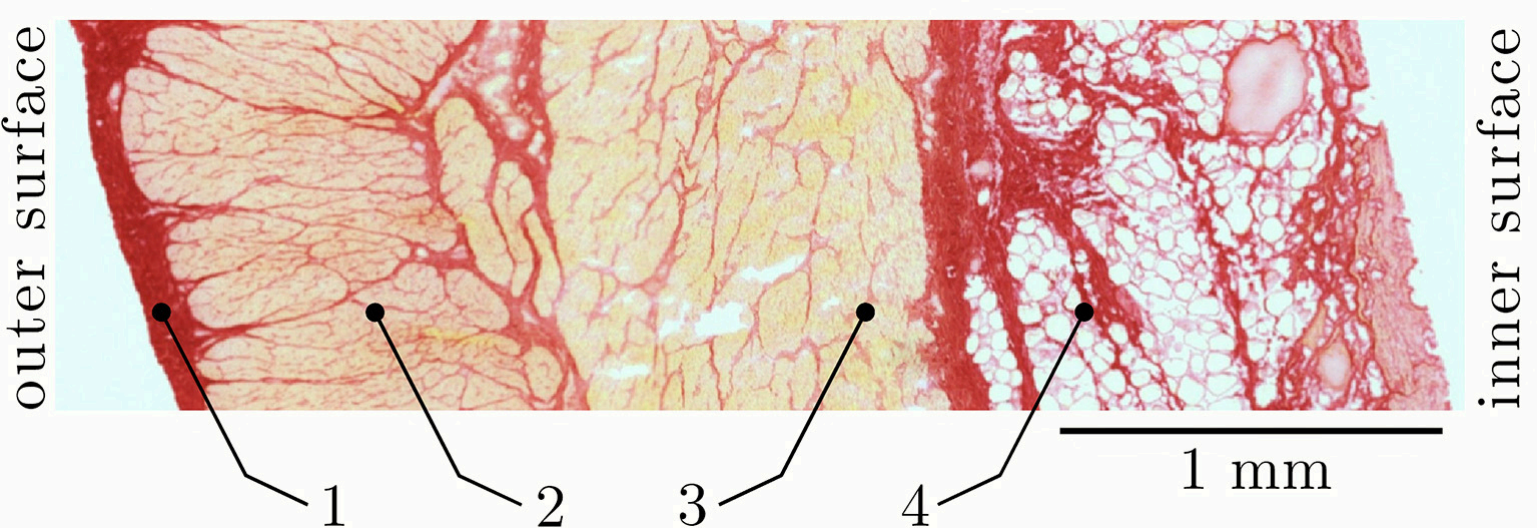
Section of a characteristic smooth tissue sample from porcine stomach. Representative picture from histological staining of samples from the proximal stomach (fundus) at 200% slack length (*L*_*S*_) in longitudinal direction. Four distinct layers can be identified: (1) tunica serosa, (2) longitudinal muscle layer (tunica muscularis), (3) transversal (circumferential) muscle layer (tunica muscularis), and (4) tela submucosa.

The histological examination was carried out in accordance with the procedure previously described by Menzel et al. (2017) and has been realized on samples of the same tissue-region that were used for the dynamic parameter-determination in the present study. Briefly, the cryo-histological sections of smooth gastric tissue strips were stained with Picrosirius Red staining protocol (Junqueira et al., 1979) at 200% *L*_*S*_, and photographed using a digital microscope (Zeiss Smartzoom 5). To examine the muscle cross-sectional areas (CSA) in the longitudinal direction (Figure 1), the average length and width of the individual sample strips (Figure 3) was determined with an image editing software (ImageJ 1.49 v, National Institutes of Health, USA). The CSA was calculated by assuming a rectangular cross-section. The muscle tension (*P*_*im*_) of a smooth gastric tissue sample was determined with *P*_*im*_ = (*F*_*im*_/*CSA*)/*p* (where *p* describes the percentage of the longitudinal muscle layer from the total CSA).

### Data processing and statistics

The length- and force-signals from the dual-mode muscle lever were recorded at 100 Hz with an A/D interface (604A, Aurora Scientific, Canada). A real-time software package (610A Dynamic Muscle Analysis, Aurora Scientific) was used for data acquisition. A program written in Matlab (The Mathworks, Inc., Nattick, MA, USA) was used for data analysis. Data were expressed as mean ± standard deviation (*s.d*.). For statistical analyses, normalized data were used. Force values were divided by individual *F*_*im*_. Length data were divided by *L*_*s*_ and *L*_0_ and velocity data were expressed in absolute values [mm/s] and normalized to optimum muscle length [*L*_0_/s], respectively. A Kolmogorov-Smirnov Test with *P* > 0.05 indicated no deviation from normality. To test significant differences of history-effects in dependence of ramp amplitude and velocity, a one-way ANOVA with repeated measures was used. For homogeneous variances, *post-hoc* analyses were performed using the Tukey-HSD test. A significance level of *P* < 0.05 was used for all analyses. Statistical analyses were carried out using SPSS 23 (IBM Corp, Armonk, NY, USA).

## Results

### Histological characterization

The results of the structural observations of histologically stained tissue samples (ventral fundus of the stomach) at 200% slack length are illustrated in Figure 3. Based on the photographs of the histological sections different tissue types can be distinguished. Following Figure 3, the stomach wall can be divided into four distinct layers: (1) tunica serosa, (2) tunica muscularis in longitudinal- and (3) transversal (circumferential) orientation and (4) tunica submucosa. Note that the tunica mucosa is dissected from the tissue samples. All structures that stained yellow-orange correspond to muscle tissue, while the stained red structures correspond to collagen. In agreement with Zhao et al. (2010), the highest collagen content was found in the submucosal layer (in between the fat tissue). The collagen content likely determines the gastric wall stiffness since collagen is supposed to be the stress-bearing structure in most tissues (Fung, 1993; Zhao et al., 2010). Picrosirius staining also revealed a continuous intermeshing network of collagen throughout the entire stomach wall. The CSA of the longitudinal tissue strip is 30.01 ± 5.66 mm^2^ (*n* = 53), whereas the percentage of the longitudinal muscle layer from the total CSA is 36.6 ± 2.6%.

### Smooth muscle tissue properties

**Figure 4:**
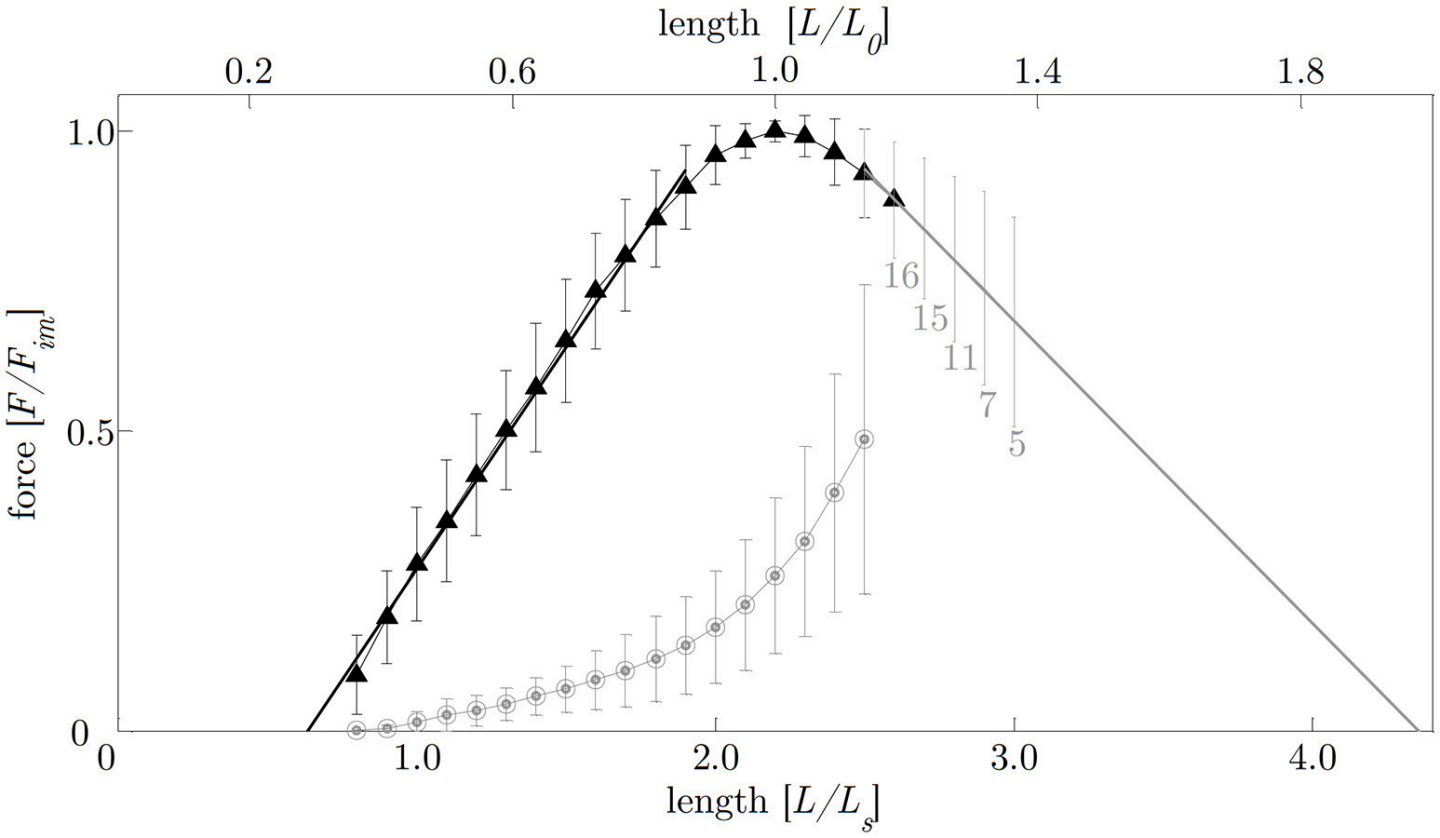
Force-length relationship. The length is normalized to optimum muscle length (*L*_0_, upper abscissa) and slack length (*L*_*S*_, lower abscissa), respectively. The force is normalized to maximum isometric force (*F*_*im*_). Filled triangles and open circles indicate mean values of active and passive isometric smooth muscle forces, respectively. Bars indicate corresponding standard deviations. The ascending limb (*f*_1_(*x*)*;* indicated by the solid black line) and descending limb (*f*_2_(*x*)*;* indicated by the solid gray line) of the force-length relation (FLR) were fitted by linear regression models following the equations:*f*_1_(*x*) = 0.74*x*−0.47 and *f*_2_(*x*) = −0.51*x*+2.2, with*x* = *L*/*L*_*S*_. Isometric force-length measurements comprise *n* = 20 tissue samples up to lengths of 2.5 *L*_*S*_ (corresponding to around 50% *F*_*im*_). For determining the descending limb of the FLR for lengths longer than 2.5 *L*_*S*_, varying sample sizes were investigated (labeled with gray numbers below standard deviation- (*s.d*.) bars on the descending limb of the FLR).

**Figure 5:**
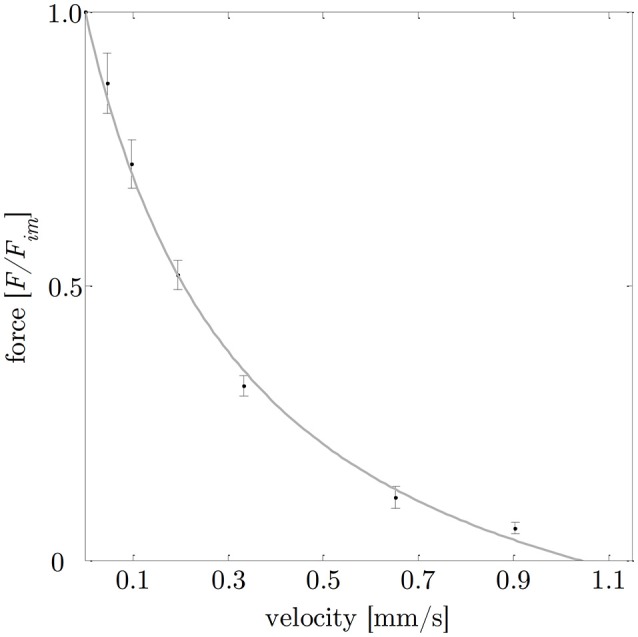
Force-velocity relationship. Based on a series of isotonic contractions, the normalized force (means ± standard deviations are indicated by black bars) was determined as function of the velocity. The gray curve shows the typical hyperbolic shape observed by Hill (1938), with $\frac{a}{F_{im}}=curv=0.36$ (*a* describes the force asymptote) and ν_*max*_ = 1.08 mm/s (the intersection of the fitted hyperbolic curve with the velocity-axis). Isotonic force-velocity measurements comprise *n* = 25 tissue samples. The force is normalized to maximum isometric force (*F*_*im*_) and the velocity is expressed in absolute values [mm/s].

The smooth gastric tissue samples exhibited a characteristic FLR—similar to skeletal muscles. The active isometric FLR showed clearly visible pronounced slope changes, characterized by a linear ascending and descending limb, and a bell-shaped plateau region (see Figure 4). The plateau region (>95% *F*_*im*_) ranged from 0.9 to 1.1 *L*_0_ or 2.0 to 2.4 *L*_*S*_. Extrapolation of the ascending and descending limb of the FLR, see section Determination of Smooth Muscle Tissue Properties, yielded zero force at 0.65 ± 0.14 *L*_*S*_ or 0.29 ± 0.06 *L*_0_ and 4.37 ± 1.00 *L*_*S*_ or 1.99 ± 0.45 *L*_0_, respectively (cf. Figure 4). The maximum isometric force (*F*_*im*_) at *L*_0_ was 1141.2 ± 295.8 mN (*n* = 20). Longitudinal muscle strips of porcine stomach, stimulated by supramaximal AC field stimulation, attained their *F*_*im*_ after 13.6 ± 1.5 s (*n* = 20) at 32°C. Based on histological observations in section Histological Characterization, the mean maximum muscle tension (*P*_*im*_) of gastric tissue samples was 10.4 ± 2.6 N/cm^2^ in longitudinal orientation. The passive FLR of the unstimulated muscle strip was characterized by an exponential increase of force with sample length, accompanied by passive forces of 25.8 ± 13% *F*_*im*_ at *L*_0_. The gastric muscle strips' ability to shorten under various loads was studied by means of a series of isotonic releases. The *V*_*max*_ was 1.08 ± 0.32 mm/s (corresponding to 0.04 ± 0.01 *L*_0_/s) with a *curv*-factor of 0.36 ± 0.15 (*n* = 25). The investigated FVR of the tested stomach samples featured the typical hyperbolic shape (see Figure 5).

**Figure 6:**
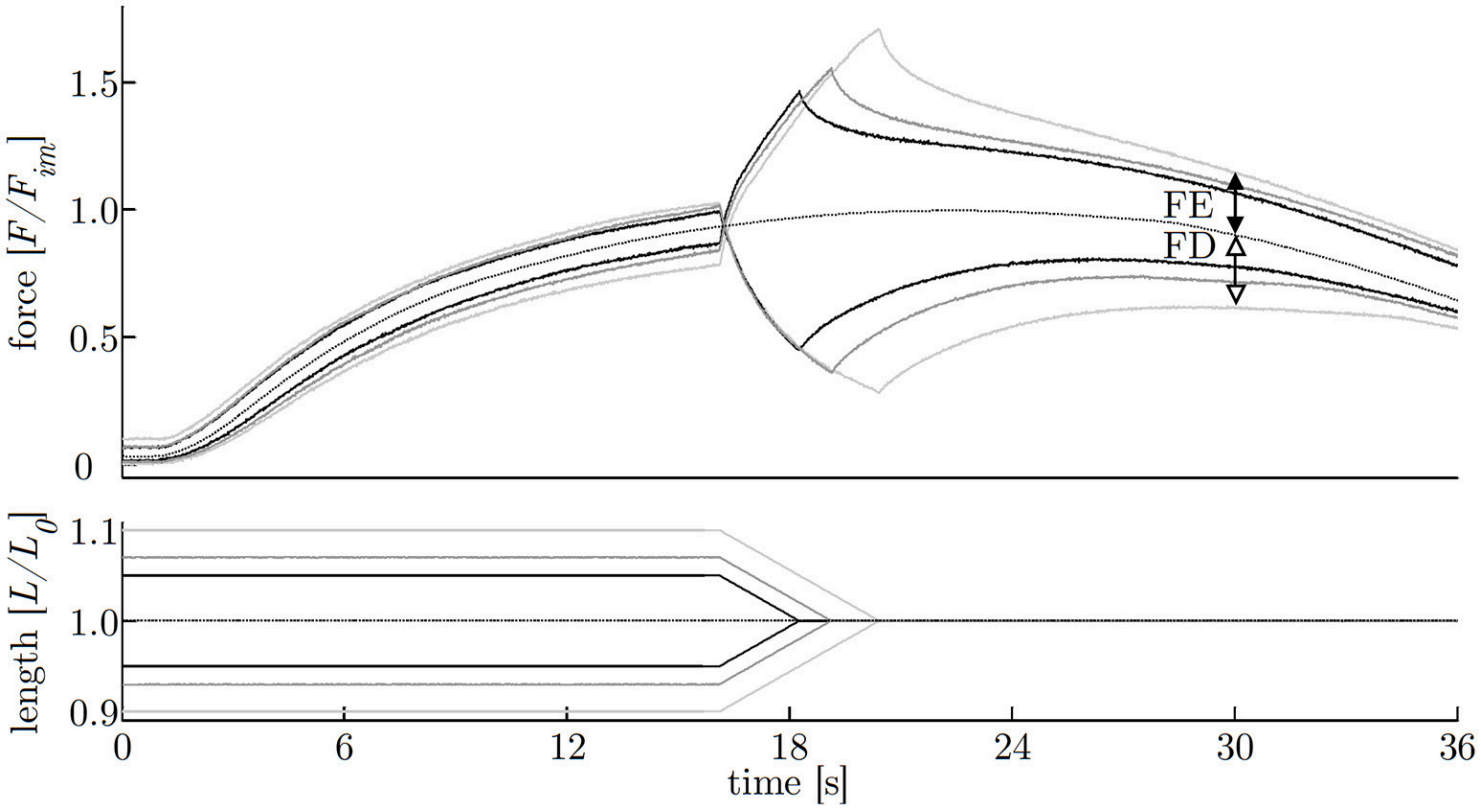
History-effects with varying ramp amplitudes. Representative force-time (upper graph) and length-time traces (lower graph) with isokinetic length changes comprising three different ramp amplitudes (5, 7, 10% *L*_0_) at constant velocity of 40% ν_*max*_ (strip number S40). The force is normalized to maximum isometric force (*F*_*im*_) and length to optimum muscle length (*L*_0_). Force enhancement (FE, difference between black arrows) and force depression (FD, difference between white arrows) are the force difference between ramp experiments (solid lines) and isometric reference contraction (dotted line) determined 10 s after the end of the ramp, shown exemplarily for the longest (10% *L*_0_) ramp.

**Figure 7:**
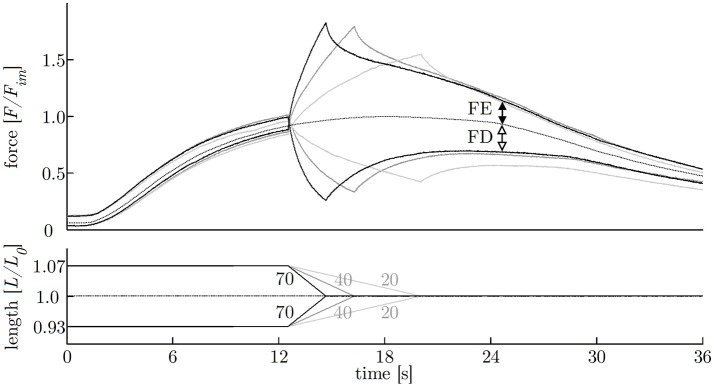
History-effects with varying ramp velocities. Representative force-time- (upper graph) and length-time traces (lower graph) with isokinetic length changes comprising three different ramp velocities (20, 40, 70% ν_*max*_) at constant ramp amplitude of 7% *L*_0_ (strip number S38); numbers without units indicate velocity in percent of maximum shortening velocity [% ν_*max*_]. The force is normalized to maximum isometric force (*F*_*im*_) and length to optimum muscle length (*L*_0_). Force enhancement (FE, difference between black arrows) and force depression (FD, difference between white arrows) are the force difference between ramp experiments (solid lines) and isometric reference contraction (dotted line) determined 10 s after the end of the ramp, shown exemplarily for the fastest (70% ν_*max*_) ramp.

**Figure 8:**
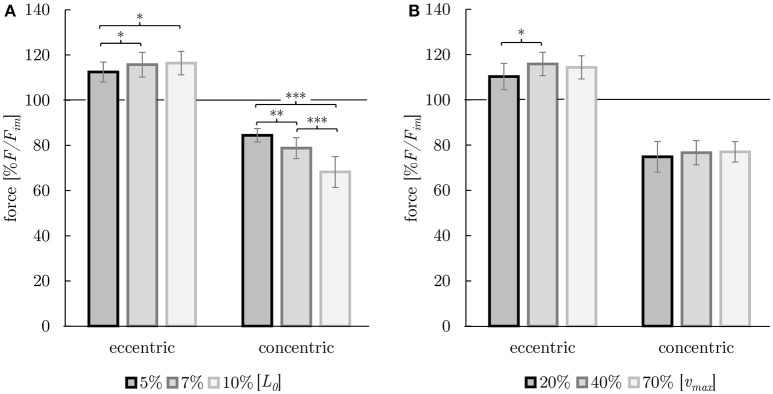
Dependency of history-effects (FE and FD) on ramp length **(A)** and on ramp velocity **(B)**. Mean forces ± standard deviations are given. Forces are normalized to the forces obtained during purely isometric reference contraction (in percent of maximum isometric force [% *F*/*F*_*im*_]) at optimum muscle length [*L*_0_] (indicated by horizontal black solid lines). **(A)** Ramp length was varied (black: 5% *L*_0_, dark gray: 7% *L*_0_, light gray: 10% *L*_0_) at constant ramp velocity (40% ν_*max*_). **(B)** Ramp velocity was varied (black: 20% ν_*max*_, dark gray: 40% ν_*max*_, light gray: 70% ν_*max*_) at constant ramp length (7% *L*_0_). Brackets and asterisks (^*^) mark differences in forces after stretch (eccentric) and shortening (concentric) in the intergroup comparison. Significance levels are marked as follows:^*^P < 0.05, ^**^P < 0.01, and ^***^P < 0.001.

The investigation of history-dependent effects yielded significantly (*P* < 0.05) enhanced forces following stretching and depressed forces following shortening compared with the corresponding isometric forces. A summary of all history-dependent effects investigated in this study is shown in Table 2. Statistical analyses yielded significant influence of ramp length on isometric muscle force after length change. FD and FE increased almost linearly with ramp length (see Figures 6, **8A**). FD is about twice as much (31.78% *F*_*im*_) for the longest ramp length (10% *L*_0_) compared to the shortest ramp length (15.52% *F*_*im*_ for 5% *L*_0_; *P* < 0.001) (see **Figure 8A**). For varying ramp velocities at constant ramp length (7% *L*_0_; *n* = 13) statistics yielded a significant increase in FE between slow (20% ν_*max*_) and moderate (40% ν_*max*_) stretch velocities (*P* < 0.05; cf. left columns of **Figure 8B**). Further increases in eccentric velocity up to 70% ν_*max*_ yielded no further change in FE. FD decreased by trend (from 25 to 23% *F*_*im*_) but not significantly with increasing ramp velocities (see Figure 7; cf. right columns of Figure 8B).

**Table 2:** Mean and standard deviation of enhanced forces (FE) and depressed forces (FD) determined 10 s after the end of the ramp.

	**(% *F_*im*_*)**	***n***	***P***
**FORCE ENHANCEMENT (FE)**			
Ramp length (% *L_0_*)			
5	12.45 ± 4.39	10	0.05 (#†)
7	15.68 ± 5.43	10	
10	16.38 ± 5.21	10	
Ramp velocity (% *ν_*max*_*)			
20	10.28 ± 5.81	13	0.01(Δ)
40	15.83 ± 5.2	13	
70	14.37 ± 5.12	13	
**FORCE DEPRESSION (FD)**			
Ramp length (% *L_0_*)			
5	15.52 ± 3.01	10	0.001(#)
7	21.23 ± 4.65	10	0.00(†*)
10	31.78 ± 6.81	10	
Ramp velocity (% *ν_*max*_*)			
20	25.16 ± 6.78	13	*ns*
40	23.35 ± 5.33	13	
70	23.00 ± 4.53	13	

History-effects were determined for three different ramp amplitudes (5, 7, 10% L_0_) and three varying velocities (20, 40, 70% ν_max_). Significant differences (P < 0.05) are marked as follows:

# between 5 and 7% L_0_,

† *between 5 and 10% L_0_*,

* between 7 and 10% L_0_,

Δ *between 20 and 40% ν_max_. ns means “not significant” and n is the number of samples*.

## Discussion

A comprehensive data set consisting of histological as well as specific biomechanical muscle properties (such as force-length and FVRs has been investigated within this study. Furthermore, this study represents the first *in-vitro* approach that examined the influence of history-dependent effects induced by ramps with various lengths and velocities on stomach smooth muscle force.

### Smooth gastric muscle properties—comparison with the literature

Mean maximum tension of active smooth muscle from porcine stomach is 10.92 ± 2.85 N/cm^2^. This value is about 2–3 times higher compared to muscle tension values of porcine bladder tissue (2.5–6.0 N/cm^2^ van Mastrigt and Glerum, 1985; Menzel et al., 2017), but about half as much when compared to smooth muscle tissue of stomachs from small mammals such as guinea pigs (19.2 N/cm^2^ Moriya and Miyazaki, 1982). The mean passive forces of about 26% *F*_*im*_ at *L*_0_ are in accordance with previous findings on smooth muscle tissue (Gordon and Siegman, 1971; Siegman et al., 2013; Menzel et al., 2017).

In agreement with the FLR from porcine urinary bladder (Menzel et al., 2017), rabbit taenia coli (Gordon and Siegman, 1971), and rat arterial vessel (Mulvany and Warshaw, 1979), the force-length dependency determined in the present study exhibited an obvious linearity of the ascending and the descending limb (see Figure 4). Furthermore, the shape of the active FLR of smooth muscles is generally similar to that of striated skeletal muscles (Gordon et al., 1966; Herlihy and Murphy, 1973)—implicating a dependence of muscle force with regards to myofilament overlap and muscle length. Considering this, active force production in smooth muscles is roughly based on the mechanisms described by the sliding filament and cross-bridge theories (Huxley and Hanson, 1954; Huxley and Niedergerke, 1954; Gordon et al., 1966) for skeletal muscles, although this is much more thoroughly understood in smooth muscles (Gordon and Siegman, 1971; Arner and Malmqvist, 1998; Siegman et al., 2013). However, there are distinct differences in the underlying microstructure of smooth muscles. Actin filaments are connected to dense bodies (Somlyo et al., 1973) and thus there is no *Z*-disc as in striated muscles. In contrast to the perfect straight alignment of actin and myosin filaments in skeletal muscles, ultrastructural studies of smooth muscles demonstrate a quite randomly orientated arrangement of myofilaments under activation, accompanied by cellular twisting (corkscrew-like) during active shortening (Fay and Delise, 1973; Bond and Somlyo, 1982; Warshaw et al., 1987).

These structural characteristics might explain specific differences in the muscle properties between smooth and striated muscles. Smooth muscles exhibit no slope change at the ascending limb of the FLR (Herrera et al., 2005), which is typical for striated muscles (Rode and Siebert, 2009; Winters et al., 2011) and muscle fibers (Stephenson, 2003; Tomalka et al., 2017) at sarcomere lengths of about 1.7 μm. In striated muscles this slope change is attributed to the myosin filament sliding through the *Z*-disc (Rode et al., 2016). The absence of this slope change in smooth muscles (Figure 4) might be partially explained by the side-polar myosin filaments in smooth muscles (Herrera et al., 2005) compared to bipolar myosin filaments in striated muscles (Craig and Megerman, 1977). However, the structural understanding of the entire FLR in smooth muscles is incomplete so far (Siegman et al., 2013).

The maximal shortening length of smooth gastric muscle tissue from pigs investigated in this study is 0.65 ± 0.14 *L*_*S*_ (corresponding to 0.29 ± 0.06 *L*_0_), which is in accordance with findings of other smooth muscle studies (Gordon and Siegman, 1971; Mulvany and Warshaw, 1979; Siegman et al., 2013). The descending limb is characterized by a linear force decrease in proportion to increasing length. This yielded zero forces at 4.37 ± 1.0 *L*_*S*_ or 1.99 ± 0.45 *L*_0_, accompanied by non-myofilament overlap. These values are slightly higher than those for such smooth muscles as rat arterial vessel (1.82 *L*_0_) (Mulvany and Warshaw, 1979) or rabbit taenia coli muscles (~1.9 *L*_0_) (Siegman et al., 2013). For lengthening skeletal muscles, active force production is limited to about 1.6 *L*_0_ (Gordon et al., 1966; Stephenson, 2003). Differences might be due to the functional necessity of gastric tissue to withstand high distension (Korossis et al., 2009; Jia et al., 2015). The complex microstructure of smooth muscles comprises a loose, irregular myofilament arrangement held in a mesh, whereas intermediate filaments appear to link the dense bodies in a cytoskeletal network (Fay and Delise, 1973; Somlyo et al., 1973; Mulvany and Warshaw, 1979; Bond and Somlyo, 1982). These filaments, which have a more structural rather than a contractile function, are assumed to have a role in force transmission and mechanical stability (Arner and Malmqvist, 1998; Tortora and Nielsen, 2013). Additionally, smooth muscles are able to actively contract in response to stretching, followed by a reduction in tension within a short period of time (Hill, 1926; Gordon and Siegman, 1971; Tortora and Nielsen, 2013). This stress-relaxation response allows smooth muscles to undergo great length changes while still preserving the ability to contract efficiently (Siegman et al., 1976; Tortora and Nielsen, 2013). Furthermore, side-polar myosin filaments could help to explain the ability of smooth muscles to shorten by large amounts (Xu et al., 1996).

The *curv*-factor (0.36 ± 0.15) observed in this study is within the range (0.1–0.5) reported for smooth- (Moriya and Miyazaki, 1985; van Mastrigt, 2002; Menzel et al., 2017) and skeletal muscles (Siebert et al., 2015), respectively. Maximum shortening velocity (0.04 ± 0.01 *L*_0_/s) is in the lower range of values from 0.03– 0.6 *L*_0_/s reported for other mammalian smooth muscles (Gordon and Siegman, 1971; Kong and Stephens, 1983; Moriya and Miyazaki, 1985; van Mastrigt, 2002; Menzel et al., 2017). Compared to values reported for skeletal muscles (ν_*max*_: 3–7 *L*_0_/s, Ranatunga, 1984; Siebert et al., 2008; Gollapudi and Lin, 2013), maximum shortening velocities of smooth muscles were one to two orders of magnitude smaller (Barany, 1967).

The behavior of the smooth gastric tissue strips investigated in this study is generally in agreement with history-effects reported for other smooth- (Gunst, 1986; van Asselt et al., 2007; Menzel et al., 2017) and skeletal muscles (Rassier and Herzog, 2004; Siebert et al., 2015). Whereas, the evidence of FD and FE has been shown for other canine and porcine smooth muscles in previous studies (Gunst, 1986 trachea; van Asselt et al., 2007; Menzel et al., 2017 urinary bladder), this study represents the first approach investigating smooth gastric muscle tissue. Findings reveal a linear dependency of history-effects (FE and FD) with regards to their ramp amplitude (Table 2, Figure 6) similar to findings on skeletal muscles (Abbott and Aubert, 1952; Edman et al., 1982). Maximum values of FE (up to 16% *F*_*im*_) and FD (up to 32% *F*_*im*_) exceed previous findings on smooth muscles (12 and 18% *F*_*im*_ for FE and FD, respectively, Menzel et al., 2017).

In accordance with previous investigations (Menzel et al., 2017), ramp velocity was found to have no significant influence on FD and FE, except for differences between lengthening contractions with 20 and 40% ν_*max*_ (*P* < 0.05; Table 2). The investigated influence of ramp velocity on FE (at low ramp velocities) might be influenced by the experimental protocol and the contractile properties of smooth gastric muscle tissue. Low ramp velocities require long stimulus durations resulting in a decrease in muscle force induced by fatigue—accompanied by an additional flattening of the force trace—yielding to significantly reduced FE for slow eccentric ramps (Table 2, Figure 8B). Thus, results of FE for low velocities should be considered with caution. In accordance with findings by Menzel et al. (2017) on smooth muscle tissue as well as on skeletal muscles (Abbott and Aubert, 1952), FD decreased by trend (but not significantly) with increasing ramp velocity.

### Underlying mechanisms of history-dependence of muscle force

To date, there is an intensive debate about mechanisms and functions of history-dependent effects in skeletal muscles (Herzog et al., 2008; Campbell and Campbell, 2011; Siebert and Rode, 2014; Hessel et al., 2017). Titin, a non-cross-bridge, semi-active structure —is increasingly recognized as an important protein that contributes to active force production during and following eccentric contractions (Herzog et al., 2016). While titin does only exist in striated muscles, a molecule having similar functional and structural titin-like characteristics, named *smitin*, occurs in smooth muscles (Kim and Keller, 2002). Therefore, a transfer of currently discussed various mechanisms, that provide some possible explanations with regards to history-dependent effects in skeletal muscles, is likely (Rode et al., 2009; Nishikawa et al., 2012; Schappacher-Tilp et al., 2015; Heidlauf et al., 2016). However, further experimental and modeling evidence is required to demonstrate conclusive explanatory approaches of underlying mechanisms of history-dependent effects of force production in smooth muscle tissue (see Supplementary Material for further information).

### Functional and physiological relevance

The investigated tissue samples were dissected out of a predefined section of the fundus (see Figure 1, black rectangle), which is mainly serving as food reservoir. Hence, when the proximal stomach is filled due to ingestion, accompanied by a large expansion of surrounding tissue, history-dependent effects, observed experimentally in this study, might have physiological relevance in order to suit gastric motility. The motility of the fundus (proximal stomach) is characterized by almost tonic contractions induced by permanent but irregular muscle activity (de Wever et al., 1978; Azpiroz and Malagelada, 1984; Notivol et al., 1995). More specifically, in between meals (interdigestive phase) the fundus maintains a high basal muscle tone (a state of continuous partial contraction). Upon food intake, the muscle tone of the proximal stomach suppresses, but will never be switched off completely (Notivol et al., 1995; Schwizer et al., 2002; Tack et al., 2002; Janssen et al., 2011). This enables gastric accommodation and enhances the storage capacity of the stomach by increasing the compliance of the fundic stomach muscle (Villanova et al., 1997; Kindt and Tack, 2006). These findings suggest an eccentric contraction behavior of the fundus during ingestion, even though at suppressed gastric tone (Azpiroz and Malagelada, 1984; Janssen et al., 2011). Accordingly, we expect enhanced forces following active muscle stretch which might subsequently support gastric emptying. This potentially explains the functional relevance of significantly enhanced forces during and after lengthening as observed in this study. Hence, a protective and supportive function of FE prior to gastric emptying to avoid excessive distension of the stomach might represent specific adaptations to gastric functionality. Furthermore, FE might counteract rapid extension of gastric tissue due to acceleration of gastric contents in impact situations e.g., at ground contact during jumping or tumbling. Additionally, the large working range of about 3.7 *L*_*S*_, high muscle tension of smooth gastric tissue, as well as the ability to withstand large eccentric forces, enables the fundus to operate suitably during filling.

FD might occur following food consumption (postprandial phase), when the fundus propels the gastric content distally by a tonic concentric contraction, going along with considerably dimensional changes accompanied by slow muscle shortening (Schulze-Delrieu et al., 1998; Farré and Tack, 2013). Anyway, high FD values seem to be functionally counterproductive for this region and could be seen as an unwanted by-product of FE (Rode et al., 2009). Consequently, the observed muscle properties of the fundus muscle tissue might represent functional adaptations to cover prevailing conditions. However, to verify the hypothesized relation between contractile properties and stomach function, structural reasons for history-dependent effects in smooth muscles as well as potential mechanisms of adaptation have to be analyzed in prospective studies.

Concluding, the findings provided by the current research support the idea of a holistic reflection of stomach structure and function, extending well-known mechanisms and processes (such as distinct signal pathways, various triggered mechanoreceptors) by history-dependent effects. A key role in explaining the enhanced and depressed total force-responses during active stretching and shortening, respectively, might represent the titin-like structure *smitin*. This semi-active element might behave like a spring during fundic distension and emptying, thus energy may be stored and recoiled as reported for skeletal muscles in stretch-shortening cycles (Roberts and Azizi, 2011), respectively. Consequently, an elegant, energy saving, elastic mechanism similar to the bouncing gait in vertebrate locomotion (Cavagna et al., 1977; Roberts and Azizi, 2011; Roberts, 2016) might enable, or at least enhance, efficient stomach function. The results presented here enable new insights into stomach function and might facilitate development and validation of realistic 3D muscle models of hollow organs like the stomach.

## Author contributions

TS, MBö and AT conceived and designed the experiments. AT and MBo performed the experiments; AT, MBo, and TS analyzed the data. AT prepared the figures; TS, AT, and MBo interpreted results; TS, AT, and MBö edited, revised, and drafted the manuscript; all authors approved the final version of manuscript.

### Conflict of interest statement

The authors declare that the research was conducted in the absence of any commercial or financial relationships that could be construed as a potential conflict of interest.

## Abbreviations

CSA: cross-sectional area
*curv*: curvature factor
*F*_*im*_: maximum isometric muscle force
FD: force depression
FE: force enhancement
FLR: force-length relation
FVR: force-velocity relation
*L*_0_,: optimum muscle length associated with *F*_*im*;_ *L*_*S*_ slack length
*p*: percentage of longitudinal muscle layer from total CSA
*P*_*im*_: maximum smooth muscle tension
ν_*max*_: maximum shortening velocity.
