# Supplementary material for: Porcine Stomach Smooth Muscle Force Depends on History-Effects
Source: Front Physiol. 2017 Oct 18;8:802. doi: 10.3389/fphys.2017.00802 (PMC5651592; doi:10.3389/fphys.2017.00802)
Supplement: Supplementary file 1 [file DataSheet1.DOCX]

Supplementary Material

**Porcine stomach smooth muscle force depends on history-effects**

André Tomalka^*^, Mischa Borsdorf, Markus Böl, Tobias Siebert

*** Correspondence:** André Tomalka: [andre.tomalka@inspo.uni-stuttgart.de](mailto:andre.tomalka@inspo.uni-stuttgart.de)

**‘History-effects and their relevance on smooth muscle modelling’**

To date, the contribution of titin – a semi-active, non-cross-bridge structure in striated muscles – seems to a have a key role in explaining particularly enhanced total forces during and after active stretching in striated muscles (Leonard and Herzog, 2010; Monroy et al., 2017; Powers et al., 2014; Rode et al., 2009; Shalabi et al., 2017; Tomalka et al., 2017). While the structural protein titin does only exist in striated muscles, a molecule having similar functional and structural titin-like characteristics, named *smitin*, occurs in smooth muscles (Kim and Keller, 2002). Anyhow, a conclusive understanding of underlying titin-actin mechanisms in striated muscles (or of the potential smitin-actin interaction in smooth muscles), is still lacking and remains highly speculative (Menzel et al., 2017; Shalabi et al., 2017; Siebert and Rode, 2014). Alternatively, it has been hypothesised that classic cross-bridge dynamics may be modified during eccentric contractions in skeletal muscles (Walcott and Herzog, 2008). Similar behaviour has been experimentally described for smooth muscles by Dillon et al. (1981) and Arner and Malmqvist (1998), whereby cross‑bridges enter the so-called ‘latch state’. The rate of detachment in this state might depend on the strain of cross-bridges. Thus, to cover prevailing mechanical conditions, high loads lead to prolonged binding of cross-bridges, and low loads accelerate cross-bridge release (Huxley and Simmons, 1971; Veigel et al., 2003). This dynamic modification of contractile properties in smooth muscle tissue might lead to shifts of contractile phenotypes (’slow‘ to ’fast‘ and vice versa) following small length-changes or hormonal-adaptations (Arner and Malmqvist, 1998). However, the dynamic shifts of the contractile properties seem to require several days (Arner and Malmqvist, 1998), and the ‘stuck cross-bridge’ idea could not be validated in experiments, neither on skeletal muscles (Mehta and Herzog, 2008) nor on smooth muscles (Butler et al., 1983). Hence, these theories are unlikely explanations for force enhancement at pronounced stretches. Further experimental and modelling evidence is necessary to demonstrate conclusive explanatory approaches of underlying mechanisms of history-dependent effects of force production in smooth muscle tissue.

Hence, the need for realistic, experimental input data with regards to electrical-, chemical- and biomechanical properties (Böl et al., 2015) becomes more and more important for the development and validation of 3D muscle models (Böl et al., 2011a; Böl et al., 2011b; Heidlauf et al., 2016; Röhrle et al., 2016). These models potentially provide a detailed insight in the functionality and motility of hollow organs (e.g. bladder (Seydewitz et al., 2017) and arteries (Böl and Schmitz, 2013; Böl et al., 2012; Schmitz and Böl, 2011)), contribute to a deeper understanding of pathophysiological changes and diseases, and might be used to assess specific treatments (Cheng et al., 2007; Cheng et al., 2010).

In addition to the general enhancement of the physiological understanding of history-effects in smooth muscle tissues, the presented data can also be helpful for the development of more comprehensive, numerical models. Traditional smooth muscle models that are able to simulate the active excitation behaviour of smooth muscle tissue (Böl and Schmitz, 2013; Böl et al., 2012; Cheng et al., 2007; Cheng et al., 2010; Schmitz and Böl, 2011; Seydewitz et al., 2017) do not consider history-effects. However, the consideration of those effects by implementing them into modelling approaches would increase the prediction accuracy. Beside this, for realistic model validation information such as, for example, layer-specific active and passive tissue characteristics, location-dependent fibre orientation, and data about the innervation characteristics, are still missing.

Hence, further structural and biomechanical properties will be needed as input data for three-dimensional stomach models. In future, validated models should be capable of predicting varying functional effects of pathological tissue alterations (e.g. due to certain diseases such as cancer). Building a computational stomach model might assist in the training, the preparation and the planning of complex surgery of the gastrointestinal tract (e.g. by using a virtual endoscopic navigation system (Gastelum et al., 2016)) due to the visualisation and navigation through anatomical structures (Hochberger et al., 2002). Moreover, models potentially enable the simulation of dynamic behaviour (*peristalsis*) and help to understand certain physical responses and pathophysiological abnormalities as e.g. vomiting (*emesis*) and *pylorospasm* (Horn, 2008).

**References**

**Arner, A. and Malmqvist, U.** (1998). Cross-bridge cycling in smooth muscle: a short review. *Acta Physiol Scand.* **164**, 363–372.

**Böl, M. and Schmitz, A.** (2013). A coupled chemomechanical model for smooth muscle contraction. In *Computer Models in Biomechanics, From Nano to Macro.* (ed. Holzapfel, G.A. & Kuhl, E.), pp. 63–75. Springer Netherlands.

**Böl, M., Sturmat, M., Weichert, C. and Kober, C.** (2011a). A new approach for the validation of skeletal muscle modelling using MRI data. *Comput. Mech.* **47**, 591–601.

**Böl, M., Weikert, R. and Weichert, C.** (2011b). A coupled electromechanical model for the excitation-dependent contraction of skeletal muscle. *J. Mech. Behav. Biomed. Mater.* **4**, 1299–1310.

**Böl, M., Schmitz, A., Nowak, G. and Siebert, T.** (2012). A three-dimensional chemo-mechanical continuum model for smooth muscle contraction. *J. Mech. Behav. Biomed. Mater.* **13**, 215–229.

**Böl, M., Leichsenring, K., Ernst, M., Wick, C., Blickhan, R. and Siebert, T.** (2015). Novel microstructural findings in M. plantaris and their impact during active and passive loading at the macro level. *J. Mech. Behav. Biomed. Mater.* **51**, 25–39.

**Butler, T. M., Siegman, M. J. and Mooers, S. U.** (1983). Chemical Energy Usage During Shortening and Work Production in Mammalian Smooth-Muscle. *Am. J. Physiol.* **244**, C234–C242.

**Cheng, L. K., Komuro, R., Austin, T. M., Buist, M. L. and Pullan, A. J.** (2007). Anatomically realistic multiscale models of normal and abnormal gastrointestinal electrical activity. *World J. Gastroenterol.* **13**, 1378–1383.

**Cheng, L. K., O’Grady, G., Du, P., Egbuji, J. U., Windsor, J. A. and Pullan, A. J.** (2010). Gastrointestinal system. *Wiley Interdiscip. Rev. Syst. Biol. Med.* **2**, 65–79.

**Dillon, P. F., Aksoy, M. O., Driska, S. T. and Murphy, R. A.** (1981). Myosin phosphorylation and the cross-bridge cycle in arterial smooth muscle. *Science* **211**, 495–497.

**Gastelum, A., Mata, L., Brito-de-la-Fuente, E., Delmas, P., Vicente, W., Salinas-Vázquez, M., Ascanio, G. and Marquez, J.** (2016). Building a three-dimensional model of the upper gastrointestinal tract for computer simulations of swallowing. *Med. Biol. Eng. Comput.* **54**, 525–534.

**Heidlauf, T., Klotz, T., Rode, C., Altan, E., Bleiler, C., Siebert, T. and Röhrle, O.** (2016). A multi-scale continuum model of skeletal muscle mechanics predicting force enhancement based on actin–titin interaction. *Biomech. Model. Mechanobiol.* **15**, 1423–1437.

**Hochberger, J., Maiss, J. and Hahn, E. G.** (2002). The use of simulators for training in GI endoscopy. *Endoscopy* **34**, 727–729.

**Horn, C. C.** (2008). Why is the neurobiology of nausea and vomiting so important? *Appetite* **50**, 430–434.

**Huxley, A. F. and Simmons, R. M.** (1971). Proposed mechanism of force generation in striated muscle. *Nature* **233**, 533–538.

**Kim, K. and Keller, T. C. S.** (2002). Smitin, a novel smooth muscle titin-like protein, interacts with myosin filaments in vivo and in vitro. *J. Cell Biol.* **156**, 101–111.

**Leonard, T. R. and Herzog, W.** (2010). Regulation of muscle force in the absence of actin-myosin-based cross-bridge interaction. *Am. J. Physiol. Cell Physiol.* **299**, C14-20.

**Mehta, A. and Herzog, W.** (2008). Cross-bridge induced force enhancement? *J. Biomech.* **41**, 1611–1615.

**Menzel, R., Böl, M. and Siebert, T.** (2017). Importance of contraction history on muscle force of porcine urinary bladder smooth muscle. *Int. Urol. Nephrol.* **49**, 205–214.

**Monroy, J. A., Powers, K. L., Pace, C. M., Uyeno, T. and Nishikawa, K. C.** (2017). Effects of activation on the elastic properties of intact soleus muscles with a deletion in titin. *J. Exp. Biol.* **220**, 828–836.

**Powers, K., Schappacher-Tilp, G., Jinha, A., Leonard, T., Nishikawa, K. and Herzog, W.** (2014). Titin force is enhanced in actively stretched skeletal muscle. *J. Exp. Biol.* **217**, 3629–3636.

**Rode, C., Siebert, T. and Blickhan, R.** (2009). Titin-induced force enhancement and force depression: a “sticky-spring” mechanism in muscle contractions? *J. Theor. Biol.* **259**, 350–360.

**Röhrle, O., Sprenger, M. and Schmitt, S.** (2016). A two-muscle, continuum-mechanical forward simulation of the upper limb. *Biomech. Model. Mechanobiol.* **16**, 743–762.

**Schmitz, A. and Böl, M.** (2011). On a phenomenological model for active smooth muscle contraction. *J. Biomech.* **44**, 2090–2095.

**Seydewitz, R., Menzel, R., Siebert, T. and Böl, M.** (2017). Three-dimensional mechano-electrochemical model for smooth muscle contraction of the urinary bladder. *J. Mech. Behav. Biomed. Mater.* **75**, 128–146.

**Shalabi, N., Cornachione, A., Leite, F., Vengallatore, S. and Rassier, D. E.** (2017). Residual force enhancement is regulated by titin in skeletal and cardiac myofibrils. *J. Physiol.* **595**, 2085–2098.

**Siebert, T. and Rode, C.** (2014). Computational modeling of muscle biomechanics. In *Computational Modelling of Biomechanics and Biotribology in the Musculoskeletal System. Biomaterials and Tissues.* (ed. Z.Jin), pp. 173–243. 1st ed. Amsterdam: Woodhead Publishing / Elsevier.

**Tomalka, A., Rode, C., Schumacher, J. and Siebert, T.** (2017). The active force – length relationship is invisible during extensive eccentric contractions in skinned skeletal muscle fibres. *Proc. R. Soc. B Biol. Sci.* **284**, pii: 20162497.

**Veigel, C., Molloy, J. E., Schmitz, S. and Kendrick-Jones, J.** (2003). Load-dependent kinetics of force production by smooth muscle myosin measured with optical tweezers. *Nat. Cell Biol.* **5**, 980–986.

**Walcott, S. and Herzog, W.** (2008). Modeling residual force enhancement with generic cross-bridge models. *Math. Biosci.* **216**, 172–186.
